# VarElect: the phenotype-based variation prioritizer of the GeneCards Suite

**DOI:** 10.1186/s12864-016-2722-2

**Published:** 2016-06-23

**Authors:** Gil Stelzer, Inbar Plaschkes, Danit Oz-Levi, Anna Alkelai, Tsviya Olender, Shahar Zimmerman, Michal Twik, Frida Belinky, Simon Fishilevich, Ron Nudel, Yaron Guan-Golan, David Warshawsky, Dvir Dahary, Asher Kohn, Yaron Mazor, Sergey Kaplan, Tsippi Iny Stein, Hagit N. Baris, Noa Rappaport, Marilyn Safran, Doron Lancet

**Affiliations:** Department of Molecular Genetics, Weizmann Institute of Science, Rehovot, Israel; LifeMap Sciences Ltd, Tel Aviv, Israel; National Center for Biotechnology Information, National Library of Medicine, National Institutes of Health, Bethesda, MD 20894 USA; LifeMap Sciences Inc, Marshfield, MA 02050 USA; Toldot Genetics Ltd, Hod Hasharon, Israel; The Genetics Institute, Rambam Health Care Campus, Haifa, Israel; Rappaport School of Medicine, Technion, Haifa, Israel

**Keywords:** Variant selection, Gene prioritization, Phenotyping, Phenotype interpretation, Next generation sequencing analysis, Guilt by association

## Abstract

**Background:**

Next generation sequencing (NGS) provides a key technology for deciphering the genetic underpinnings of human diseases. Typical NGS analyses of a patient depict tens of thousands non-reference coding variants, but only one or very few are expected to be significant for the relevant disorder. In a filtering stage, one employs family segregation, rarity in the population, predicted protein impact and evolutionary conservation as a means for shortening the variation list. However, narrowing down further towards culprit disease genes usually entails laborious seeking of gene-phenotype relationships, consulting numerous separate databases. Thus, a major challenge is to transition from the few hundred shortlisted genes to the most viable disease-causing candidates.

**Results:**

We describe a novel tool, VarElect (http://ve.genecards.org), a comprehensive phenotype-dependent variant/gene prioritizer, based on the widely-used GeneCards, which helps rapidly identify causal mutations with extensive evidence. The GeneCards suite offers an effective and speedy alternative, whereby >120 gene-centric automatically-mined data sources are jointly available for the task. VarElect cashes on this wealth of information, as well as on GeneCards’ powerful free-text Boolean search and scoring capabilities, proficiently matching variant-containing genes to submitted disease/symptom keywords. The tool also leverages the rich disease and pathway information of MalaCards, the human disease database, and PathCards, the unified pathway (SuperPaths) database, both within the GeneCards Suite. The VarElect algorithm infers direct as well as indirect links between genes and phenotypes, the latter benefitting from GeneCards’ diverse gene-to-gene data links in GenesLikeMe. Finally, our tool offers an extensive gene-phenotype evidence portrayal (“MiniCards”) and hyperlinks to the parent databases.

**Conclusions:**

We demonstrate that VarElect compares favorably with several often-used NGS phenotyping tools, thus providing a robust facility for ranking genes, pointing out their likelihood to be related to a patient’s disease. VarElect’s capacity to automatically process numerous NGS cases, either in stand-alone format or in VCF-analyzer mode (TGex and VarAnnot), is indispensable for emerging clinical projects that involve thousands of whole exome/genome NGS analyses.

**Electronic supplementary material:**

The online version of this article (doi:10.1186/s12864-016-2722-2) contains supplementary material, which is available to authorized users.

## Background

There is increasing recognition by the scientific community that next generation sequencing (NGS) is a pivotal technology for diagnosing the genetic cause of many human diseases. This acknowledgment is taking form in several large-scale projects which implement NGS as a key instrument for elucidating the genetic components of rare diseases and cancer [1]. Other clinical studies aimed at deciphering monogenic and complex diseases have also demonstrated the effectiveness of various NGS approaches such as whole genome sequencing, whole exome and gene panel sequencing, as exemplified in [2–6].

Primary analysis of disease NGS results includes sequence read mapping and variant calling, with results stored in a Variant Call Format (VCF) file. The VCF file typically contains ~20,000-50,000 positions that differ from the reference genome (“variant long list”). Subsequently, analysis pipelines sift these SNPs and indels by populating the VCF file with annotation data, such as segregation in affected families, genetic linkage information [7], population frequency [8] and missense protein impact as exemplified by PolyPhen [9] SIFT [10] and SNAP2 [11], all facilitating variant filtration (secondary analysis). This helps generate a “variant medium list”, with a few dozen to a few hundred entries, depending on the assumed mode of inheritance and on the employed filtering cutoffs.

In the foregoing analyses, variants are analyzed without regard to the disease phenotype of the sequenced individual. As a first step in introducing phenotype relationships, many pipelines utilize knowledge on variant-disease relations (e.g. ClinVar [12] or COSMIC [13]) for further filtration of the sequence variants.

A typical gene can have a multitude of variants that have not yet been documented to have a relationship with a disease or a phenotype. So in most cases, none of the annotated variant-disease relations appear relevant to the sequenced subject. Furthermore, it has been shown that every genome harbors about 100 loss of function variants with about 20 genes completely inactivated [14], so frequency and impact filtering is not sufficient to identify causative variants. To address these challenges, a gene-based interpretation process becomes necessary. The strategy entails finding disease or phenotype relationships for the gene and not for the variant contained within it.

**Fig. 1:**
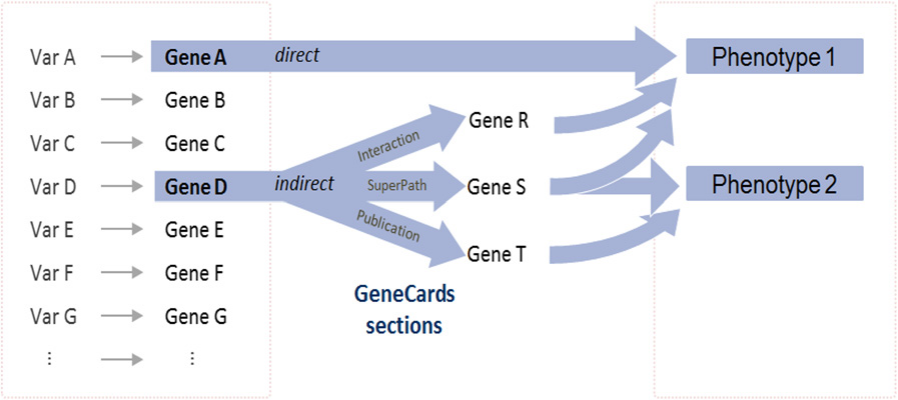
VarElect information flow. Variants-containing genes are mapped by VarElect, in an example, to two OR-ed phenotype keywords. In the direct mode, Gene A is directly related to phenotype 1. In the indirect mode, Gene D is related to both Phenotype 1 and Phenotype 2. The three implicating genes R, S and T generate Gene-Gene-phenotype connections as shown via three GeneCards sections

In this paper, we describe the construction and use of VarElect (ve.genecards.org), a free web-based phenotype-dependent NGS variant prioritizer, which leverages the wealth of information in GeneCards and its affiliated databases [15–19] (Fig. 1). VarElect employs GeneCards’ powerful search and scoring capacities, and its algorithm affords inferring direct as well as indirect links between sequenced genes and disease/symptom/phenotype keywords. The indirect links benefit from GeneCards’ excellent capacity to relate genes to each other via numerous annotations. VarElect thus provides a robust facility for ranking genes and pointing out their likelihood to be related to a patient’s disease.

## Methods

### Scoring

The scores included in VarElect results basically stem from Elasticsearch technology [20]. The score of a specific term is determined by the frequency of its appearance in the individual GeneCard, as compared to that in all genes (inverse document frequency correction). While Lucene-based search mechanisms such as Elastic and Solr might cause various biases, e.g. dependence on how in-depth a topic is studied and published, Solr search is commonplace in semantic retrieval and navigation in scientific document collections (cf [21]). Further, boosting factors are applied in VarElect for important sections (e.g. Disorders or Function) reflecting the greater significance attributed to gene-phenotype connections found via these annotations. For Boolean multi-word searches, the complete query is first sent to the search engine to determine the set of genes that match the complete Boolean query. For such genes, the query is then broken into search terms (single word or quoted multiple words), each scored independently, and the individual scores are summed.

In the indirect mode, scores include both the strength of the relationship AB between gene A (implicated) and gene B (implicating) as well as the strength of the relationship BP between gene B and phenotype P. The former scores are obtained from a pre-computed gene-to-gene matrix, constructed based on all pairwise gene-symbol search relationships in the GeneCards database. This indicates the propensity of gene A’s symbol within the GeneCard of gene B, e.g. in summaries, publications, pathways, interactions or paralogy relations. The final score is computed by:

$$ \mathrm{F}\cdot {\displaystyle \sum_{i=1}^5{2}^{1-i}A{B}_i\cdot B{P}_i} $$

The summation is for up to five B genes (cf. [22]) with the highest compound ABxBP scores. F is a normalization factor that affords a comparison between direct and indirect scores, facilitating a capacity to generate a unified (interlaced) record of direct and indirect genes. We currently use F = 0.05, a value derived by running a sample set of 500 genes with 500 different phenotype terms, of which 294 queries had both direct and indirect results, and calibrating F to show a good balance between the direct and indirect scores (Additional file 1: Figure S1).

### Interlacing of direct and indirect results

In Additional file 1: Figure S1, the score computed by the equation below, and shown as blue line, ranges from 0 to 1. The value 0 means all direct results come before all indirect results, and 1 is the reverse situation where all indirect results come before all direct results. The score is calculated by counting the number of changes needed to get the sorted score from a perfect separation (0 situation) and dividing by the maximum number of changes – the number of changes needed to reach a perfect reverse order (1 situation) - where a change is only a replacement of two adjacent results. The Formula used is:

$$ score=\left({\displaystyle \sum_{n=1}^{N={N}_D+{N}_I}\left({r}_n+{N}_D-n\right)}\right)/\left({N}_D\ast {N}_1\right) $$

where *N*_*D*_ is the number of direct items, *N*_*I*_ is the number of indirect items and *r*_*n*_ is the internal item rank, separate for the direct and indirect lists (the internal order of the direct/indirect results is not affected by the factor).

### Benchmarking queries

In order to test VarElect’s performance against Phenolyzer [23], we generated 34 benchmark queries, composed of a disease causing gene (the probe gene) and its relevant disease/phenotype/symptom terms. Of these, 13 queries were based on in-house exome analyses, 9 were based on exomes analyzed at Toldot (http://www.toldot-dna.com/content/about-us), 12 from published literature, including 4 recorded in MalaCards. In the VarElect runs, each probe gene was accompanied by background genes, some consisting of filtered gene lists from the original experiment, and others including groups of 500 randomly sampled genes from the Illumina TruSight whole exome gene panel (http://www.illumina.com/products/trusight-one-sequencing-panel.html) (see Additional file 2: Table S1 for more details).

For comparison of VarElect’s against Exomiser [24], 10 real pathological variants from solved in-house exomes were spiked (each one separately) into the demo VCF file that is distributed with Exomiser (“Pfeiffer.vcf”, after removal of the originally spiked variant). These VCF files were analyzed using hiPHIVE - an algorithm for cross-species phenotype analysis in whole-exome candidate gene prioritization [24], using the command line tool of Exomiser (V 7.1). The following parameters were used: prioritiser = hiPHIVE -F 2 -Q 30 –P true. As routinely done when using Exomiser, a restricted vocabulary tool, the submitted disease-related terms were translated to Human Phenotype Ontology (HPO) terms using Phenomizer (http://compbio.charite.de/phenomizer/). The analysis output included 715 variants from 607 genes. The same genes were submitted in parallel to VarElect using the original (untranslated) disease-related terms for gene prioritization (submission details in Additional file 3: Table S2).

For comparison of VarElect against Phevor2 [25], the same data used for Exomiser were submitted to Omicia Opal 4.16.1, using the VAAST3 filtering and prioritization pipeline [26]. Spiked VCF files were uploaded into Omicia commercial cloud application, the variants were then annotated, filtered and scored by VAAST tool version 3.0.3.6. VAAST results were further submitted for gene phenotype prioritization by Phevor 2.0.0 (with the same phenotypes as used by VarElect). The genes that passed the VAAST procedure were also submitted to VarElect. Spiked genes that were filtered out by VAAST (3 cases) were subsequently added manually to the gene list submitted to VarElect.

For comparison of VarElect against Ingenuity (IVA), the same data used for Exomiser were submitted to analysis in IVA. The IVA default filtration pipeline was used. Biological context setting was first selected as 0-hops from the relevant phenotype terms as translated by Ingenuity Pathway Analysis (IPA). If the spiked gene was not included in the list of kept genes, then the biological context filter was reset to “within 1 hop”. The gene list which included the spiked gene was submitted in parallel to VarElect using the original disease-related terms for gene prioritization in both the direct and indirect mode.

## Results and discussion

**Fig. 2:**
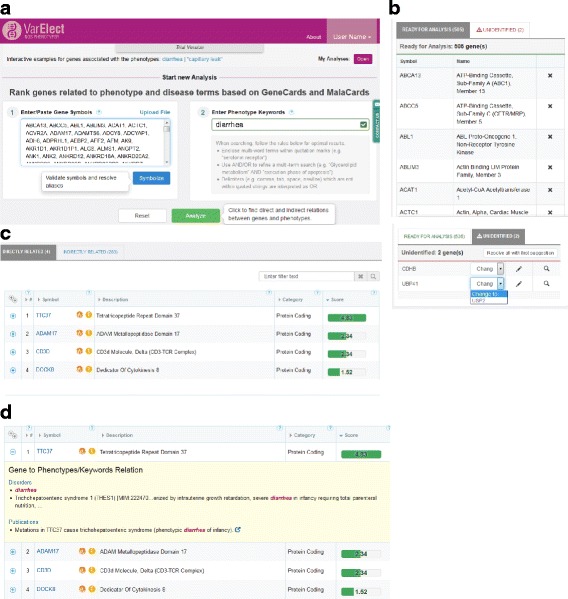
VarElect interface and direct mode example. **a** The user enters two types of input: gene symbol list and phenotype expression. A Symbolize button activates a process whereby correct symbols are assigned. The phenotype phrase undergoes automatic syntactic validation, whereby success is indicated by a green ✓ check mark. Example cases at top enable initial familiarization with the VarElect tool. “My Analyses” store previous analyses that may be accessed by the “Open” button. **b** Top, “identified” genes for which correct official symbols have been entered and are ready for analysis. Symbols may be removed at will from the input list using the X icon. Bottom, the “unidentified” tab displays cases which require symbol resolution. Suggested symbols may be selected from the drop down menu or alternate symbols may be searched for in GeneCards with the magnifying glass or entered using the edit icon. **c** Output results presented in two tabs for direct and indirect modes. Each result row displays a gene along with its basic information, such as the gene description, category and the phenotype relevance score, also indicated by the size and color of the bar. “Matched Phenotypes” and “Matched Phenotype Count” columns appear for multi-term search phrases. Results may be sorted by any of the table columns but are ranked by the relevance score by default. Gene symbols link to the relevant GeneCard, the orange icon to the variants section of the same gene and the yellow icon to a MalaCards search for the gene symbol. Entering strings within the filter text box searches for them within any of the result rows. The question marks in each column provide additional information. **d** An expanded row displays a”MiniCard” with snippets from various webcard sections, which place the exhibited evidence in context, highlighting the search words that received hits for one of the genes. External links enable further scrutiny of the information in the data sources from which it was received

VarElect’s basic functionality as a web tool entails specifying a gene symbol list imported from an experimental data file(s), together with disease phenotype and symptom terms related to the studied disorder (Fig. 2a). Genes are typically imported from a VCF file of an NGS experiment, with additional sources including lists of genes differentially expressed in transcriptome studies or those with significant variants in genome-wide association studies. VarElect then generates a scored gene list that helps the user select the best disease-causing gene candidates.

### VarElect input

In the gene entry process, one can supply a list of up to 4000 genes, with unlimited count in a version coming soon. VarElect provides a “symbolization” tool which validates that the input genes are official symbols (Fig. 2b). Whenever possible, non-symbol identifiers (i.e. aliases) are translated (with user interaction) into the corresponding official symbol. This capacity is based on the comprehensive gene alias portrayal of GeneCards [15].

The phenotype-related keywords entry is free-text, without any limitation to a closed list of terms or to an ontology. This makes it especially suited for searches of rare, unique and relatively uncharacterized medical terminology in the process of deciphering the genetic cause of the disease or exhibited symptom/phenotype. Such a capacity relies on the vast amounts of information within VarElect’s data sources in the GeneCards Suite (see VarElect knowledgebase). Phenotype entry processing incorporates real-time syntactical analysis, including a check of the Boolean logic.

Boolean searches in VarElect are especially useful in two specific scenarios, which are quite prevalent in disease NGS phenotype-based interpretation. The first is cases in which a patient is afflicted with a disorder describable by many different synonymous terms (e.g. “paraplegia”, “paraparesis”, “lower limb paralysis”, “lower extremities paralysis”). In such a case, the user would clearly wish to encompass all possibilities, and hence is advised to use an all-OR search string. The second case involves an attempt to single out a syndrome that concomitantly has several symptoms (e.g. “mental retardation”, “facial dysmorphism”, “ataxia”). In such cases, using all-AND is obviously advised.

### VarElect results and scoring

At the analysis stage, VarElect searches the bag-of-words associated with the entire GeneCard for each gene in the list, against the entered phenotype keywords. In doing so, it relies on GeneCards’ powerful search engine, and also leverages the extensive information in GeneCards. This includes key data present in the entire GeneCards knowledgebase, which automatically mines data from more than 120 sources [15, 27]. Some of the GeneCards suite-member-specific sources include MalaCards, the human disease database which encompasses ~17,000 disease entries from 15 main sources [17, 18]; PathCards, which consolidates 12 human biological pathway sources into ~1000 SuperPaths based on shared gene content [19]; and LifeMap Discovery, with its manually curated gene expression information on tissues and cells, including the realms of embryonic development and stem cells [28].

The results of VarElect analyses are shown as a table of genes with decreasing phenotype relation scores (See Methods). The gene list may also be sorted by the count of phenotype term hits in cases of multi-word search expressions. The results are shown in two separate tabs, one for genes with direct phenotype relations, and the other for those with indirect connection to the phenotype terms. The latter analyses are in the realm of “guilt by association”, whereby gene A has no connection to the phenotype, but (in an example) gene A and gene B belong to the same pathway, and gene B *is* related to the phenotype (Fig. 1, Fig. 2c). Gene B is defined as an “implicating gene” for the indirect relationships between gene A (the implicated gene) and the phenotypes.

**Fig. 3:**
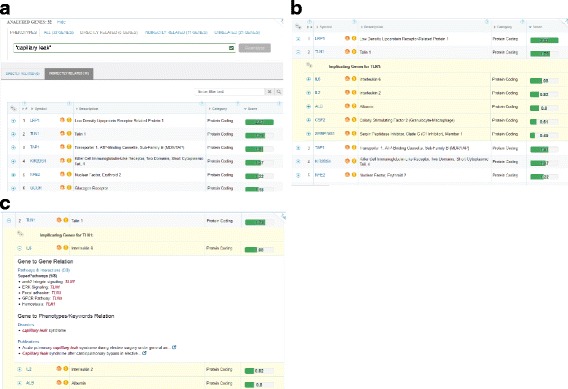
VarElect information display and indirect mode. **a** The query information section (top) displays the phenotype phrase, the fact that no direct hits were found and in a separate tab - the genes found in indirect mode. Links for un-hit genes and the entire submitted symbolized gene list are also provided. The latter may be reanalyzed by changing the phenotype phrase. **b** One of the implicated genes expanded to reveal its (top 5) implicating genes, along with their phenotype scores. **c** Implicating genes expanded to reveal two types of “MiniCards”. First - “Gene to Gene Relation”, i.e. evidence showing how the implicated gene TLN1 appears in the GeneCard for the implicating gene IL6. Second, “Gene to Phenotypes/Keywords Relation” show how the submitted phenotype keywords appear in different sections of the same implicating gene’s webcard. The provided external links enable further examination of the evidence in the source data itself

In order to enhance user insight, contextual evidence is displayed as “MiniCards”, in full analogy to such feature in GeneCards [15]. The MiniCards display the evidence for gene-keyword connection in the form of snippets from various sections of the relevant GeneCard or MalaCard [15], as well as hyperlinks to GeneCards Suite sources for further scrutiny. For direct mode, there is a single-tier MiniCards display (Fig. 2d). The indirect mode has two tiers of evidence: the first (Fig. 3a) shows up to 5 implicating genes for every implicated gene, and the second (Fig. 3c), displays MiniCards with phenotype relations for each of the 5 implicating genes.

While inter-gene score comparison is legitimate in out tool, scores resulting from different phenotype term searches cannot be readily compared at present. In an upcoming version, VarElect will include global normalization, based on the score distribution of the same phenotype expression searched within all of GeneCards. This will allow assigning a nominal *P*-value statistic to every VarElect result, even those resulting from complex Boolean queries. We will also take into account the length of the submitted gene list, analogous to multiple testing counts.

### VarElect reports and integration

#### Excel reports

VarElect exports search results into an Excel file with multiple worksheets that organize the scored gene list into direct and indirect results. In the indirect worksheet, each implicated gene is presented with several rows for its implicating genes, along with their corresponding scores. All columns are faithfully depicted in the output files, except for MiniCards which are only available via the web interface.

### TGex and VarAnnot

For users that prefer to perform the analysis in the context of a VCF file, VarElect has two alternative input–output mechanisms. The first is the TGex (Translational Genomics Expert) platform, designed to consolidate numerous annotation attributes obtained via the input of a VCF file, along with VarElect interpretation scores (based on inputted phenotypes) to a single user view with elaborate analysis parameter control. TGex generates reports in flexible formats, allowing VCF-to-clinic analyses. The second is the VarAnnot plugin, which performs a limited subset of the aformentioned analyses within a VCF-type Excel sheet. VarAnnot outputs the phenotype-gene scores into new columns. The VarElect interpretation scores in both tools can undergo joint filtering with other VCF annotation columns.

### Integration with NGS platforms

VarElect can also be integrated into primary and secondary analysis platforms or pipelines, which can benefit from enhanced phenotype interpretation capacities. This option offers both front-end and back-end application programming interfaces (APIs). The back-end API allows integrators to have better control over the interaction with VarElect within their tool, and is best leveraged when the integrating platform has secondary analysis capabilities. The front-end API allows for running a skinny version of VarElect’s web-based user interface embedded within the integrator’s analysis platform, best serving platforms that end their analysis after creating the VCF file. One such example of integration is with the Lab7 LIMS system (https://www.lab7.io/).

### VarElect knowledgebase

Herein we note some special points of strength of the GeneCards Suite databases and tools, which underlie VarElect capacities:

### MalaCards

MalaCards is a disease-centric database, with extensive information on each of >17,000 disease entries [17, 18]. Each disease is connected to a list of genes, with emphasis on a gene subset with especially strong disease relations, termed “elite genes”. Key MalaCards sections are Aliases, Summaries and Symptoms, all rich in searchable text. GeneCards and VarElect’s searches now support finding keywords in these three sections. This allows users of VarElect to perform comprehensive disease-related searches, relying on the imported MalaCards annotations. The corresponding search results are accompanied by MalaCards-based MiniCards and deep links.

### PathCards

For the benefit of VarElect searches, GeneCards contains not only pathway data [15] but also unified SuperPath information from PathCards [19]. The reason for constructing PathCards is that there is a pronounced heterogeneity in pathway naming and gene content in different original sources. To overcome this shortcoming we developed an algorithm which unifies pathways into SuperPaths [19]. This process clusters pathways based on their gene content, thus greatly reducing redundancy by integrating 3,215 pathways from 12 sources into 1,073, whilst keeping them maximally informative. An immediate outcome of SuperPath generation is the inference of new gene connections that were not reported via any pre-unification pathway, available for VarElect’s gene to gene inference.

### GenesLikeMe

GenesLikeMe, an outgrowth of GeneDecks Partner Hunter [29], creates relation metrics that quantify the sharing of common descriptors/attributes among genes. Most of these gene-gene relationships are embedded within GeneCards, thus serving the VarElect search in its indirect mode. These relations include shared pathways, shared drugs/compounds, shared protein domains, shared mouse phenotypes, shared normal tissue expression patterns (Fishilevich et al. submitted) and shared publications. Also included are relationships arising from protein-protein interaction networks and paralogy groups. GenesLikeMe entails a roadmap for future enrichment of VarElect’s indirect mode.

### VarElect advantages

#### Post-filtering phenotype interpretation

A central advantage of VarElect is its capacity to perform post-filtering gene list interpretation and scoring based on user-entered phenotype keywords. A large number of NGS analysis pipelines involve step-wise filtering based on annotation fields in a VCF file or equivalent web display. Such fields often include data from databases such as ClinVar [12] and COSMIC [13], which relate variants to disease names. This approach cannot provide an ultimate NGS solution for two reasons. The first is that not all diseases have defined names, and often a disorder is described by a combination of phenotype terms, with partial overlap to disease nomenclature. The second is that a typical gene can have thousands of variations not yet documented to have a relationship with any disease, hence in most NGS-analyzed cases, the called variations have a relatively low probability of finding variant-disease relations appearing in the disease variant repositories. VarElect’s gene centric approach and acceptance of unrestricted free text phenotype queries, combined with the power of its knowledgebase to relate terms within such queries to NGS variant-containing genes, offers a powerful enhancement to the disease-variant lookup strategy.

#### Addressing the variant-gene dichotomy

A point of strength for VarElect is its clear definition of the difference between variant-based and gene-based analyses. VarElect is defined as a ‘variant/gene prioritizer’ despite the fact that its scoring algorithms are purely gene based. The assumption is that every analyzed gene is linked to one or more variants, each with its own impact on the function of encoded protein, as listed in the annotated VCF file. When viewing the VarElect results table, a gene is considered as representative of such variant(s). As mentioned in the Background section, variant filtering will typically filter out low impact variants, first and foremost synonymous ones. Consequently, a VarElect-prioritized gene is rarely associated with multiple variants. In the event that a gene has more than one high-impact variant, the use of VarElect in its basic mode calls for going back to the VCF file to resolve the issue. In the TGex and VarAnnot modes (see the ‘VarElect reports and integration’ section) this process happens naturally as the user is presented with a variant table that includes both the variant impact and the VarElect gene prioritization score.

### Time saved

Numerous users perform NGS interpretation by manual gene by gene or semi-manual search for relationships to disease and phenotype terms. For example, this could involve scrutinizing every post-filtering gene in GeneCards, seeking connections to the appropriate keywords. Batch processing via GeneALaCart [15] affords considerable speed-up but VarElect provides a much faster and more effective solution, since it performs phenotype keyword searches in GeneCards for hundreds or even thousands of genes with a push of a button.

### Gene count flexibility

VarElect allows the user flexibility in the number of query genes, providing the freedom of different degrees of VCF filtering prior to VarElect submission.

### Extensive database

The numerous data sources mined by GeneCards and its suite members account for the myriad of links between phenotypes and genes that are often unique to VarElect.

### Powerful search engine

The Elasticsearch engine utilized by VarElect confers upon it the speed, efficacy and accuracy in relating genes to phenotype terms.

### True free-text searches

In VarElect, no restrictions are imposed on the phenotype search words used, casting a much broader search net than that afforded by tools with restricted vocabularies.

### Boolean logic

This greatly enhances VarElect’s capacity in allowing the user to choose between focusing and broadening/generalizing the search process.

### MiniCards evidence

VarElect results are displayed in MiniCards, which show in detail the phenotype terms hit contexts, along with the sections in which they reside. Hyperlinks enable quick access to detailed information in the external databases.

### MalaCards integration

MalaCards data on disease aliases, diverse summaries and disease symptom lists provide great enrichment of VarElect’s capacity for phenotype interpretation.

### Rich gene-gene links

VarElect’s indirect mode is extensively fortified by numerous sources of information affiliated with the GenesLikeMe facility. An additional strength of this mode is the capacity of VarElect to find textual appearances of a gene symbol within the entire text of a GeneCard for another gene. Finally, the interlace feature allows one to compare the strength of directly and indirectly obtained phenotype-gene relations.

### Comparison to other tools

#### Phenolyzer

**Fig. 4:**
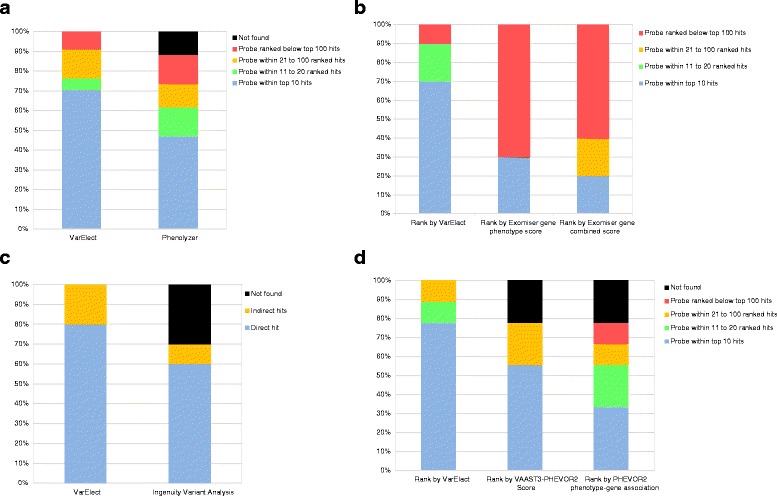
Performance comparison of VarElect to competing tools. **a** Phenolyzer: Thirty four queries were submitted to analysis in both VarElect and Phenolyzer (Methods). Each query is based on real results and includes a disease causing gene spiked into background genes, accompanied with real disease related symptoms that were recorded from the relevant patients (Table S1). The rank of the probe gene is color coded, bottom to top: top 10, top 11–20, top 21–100, below top 100, not found to be connected to the search terms. **b** Exomiser: Ten queries were submitted to both VarElect and Exomiser (Methods). The rank of the probe gene resulting from each query in Exomiser was recorded from 2 different score types; 1) Exomiser gene phenotype score that is solely derived from gene to phenotype relevance 2) Exomiser general score, combining variant severity score and gene phenotype score. Ranking results are shown with binning as in Fig. 4a. **c** Ingenuity: Queries as in Fig. 4b were submitted to Ingenuity Variant Analysis, and the appearance of the spiked gene in the resultant gene list was examined. For this comparison, Ingenuity 0-hop mode was considered comparable to VarElect direct mode, and Ingenuity 1-hop mode was considered comparable to VarElect indirect mode. **d** Phevor: Queries as in Fig. 4b were submitted to Phevor2 in combination with VAAST3 filtering (Methods). Ranking results are shown with binning as in Fig. 4a

Thirty four benchmarking queries (Methods) were submitted to both VarElect and Phenolyzer [23]. For the latter, we used the default web interface parameters, with the addition of the Mentha Protein Interaction Database. The rank of the probe gene in the output list was used for the comparison (Fig. 4a). The results show that VarElect succeeded in ranking probe genes higher than Phenolyzer. This is especially marked in cases with multi-term Boolean phenotype expressions. This advantage may be related in part to two functionality differences between the tools. The first difference is that VarElect employs strict AND logic when desired, whereby only genes found to be related to all entered terms are included in the result gene list. In order to demonstrate the strength of this option, some of the queries were entered into VarElect twice, with OR and with AND, the latter showing marked rank improvement (Additional file 2: Table S1). The second difference may be the fact that VarElect provides a free phenotype term search, while Phenolyzer utilizes a disease match algorithm for translating a submitted term to (e.g.) HPO symptoms and OMIM diseases. It appears that in general VarElect does better in Boolean query expressions with several search terms (Additional file 2: Table S1).

### Exomiser

Exomiser is a tool for functional annotation and phenotype based prioritization of variants from whole-exome sequencing data. The Exomiser-hiPHIVE algorithm combines information on the rarity of the variant and its predicted pathogenicity along with the similarity of the patient’s symptoms to animal (mouse and fish) models for each candidate gene [24]. We performed a benchmark test (Methods) examining the rank of probe genes. VarElect performed better, both when compared the “Exomiser gene phenotype score” derived from gene to phenotype relevance solely and the combined score (Fig. 4b).

### Ingenuity variant analysis (IVA)

For generating gene-phenotype relations, this tool employs a “Biological Context filter” (BCF) component. Only phenotype terms found within the IPA (Ingenuity Pathway Analysis) Knowledge Base (http://www.ingenuity.com/science/platform/content-sources) are allowed. The user has to define the relevant type of connections between the Variants/Genes and the phenotype. The user then has to browse the resulting “Path to Phenotype” network diagrams (partially analogous to VarElect’s indirect mode) for every post-filtering variant, so as to obtain user-defined relevance assessment. The IVA tool (http://www.ingenuity.com/products/variant-analysis) does not supply numerical scores to convey the strength of variant/gene-phenotype connections. VarElect’s free-text search and gene-phenotype scoring capacities thus provide clear advantages, although performing explicit benchmark comparisons to VarElect was not readily possible as IVA does not generate scores. Another advantage of VarElect over IVA is that the former involves a much higher degree of automation in the indirect mode, wherein by a push of a button, thousands of different potential functional paths are concomitantly examined without user intervention. We performed a benchmark test (Methods) based on the appearance of the target gene in the IVA filtered gene lists. VarElect performed better, both in the direct and indirect modes (Fig. 4c).

### Phevor

This tool, utilized by Omicia [25] performs phenotype interpretation via various biomedical ontologies, such as HPO, Mouse Phenotype Ontology (MPO), Disease Ontology (DO) or Gene Ontology (GO). As in VarElect’s direct mode, terms entered by Phevor users are mapped to genes that are connected to them. These relations are iteratively expanded to include terms from additional ontologies, thereupon retrieving and scoring genes farther away from the original nodes, similarly to what’s provided in VarElect’s indirect mode. Thus, VarElect and Phevor are comparable in their capacity to identify disease causing variants in genes not yet shown to be directly associated with disease. An advantage of VarElect is its reliance on a much broader, systematically mined knowledgebase in the framework of the GeneCards Suite. We performed a benchmark test (Methods) examining the rank of probe genes. VarElect performed better, both when compared to the VAAST3-PHEVOR2 scores as well as to the results of the PHEVOR2 phenotype-gene association mode (Fig. 4d).

### Clinical cases solved by VarElect

To date, VarElect helped solve about two dozen Mendelian disorders in our collaborations with Israeli medical centers. Some examples are:

#### Congenital diarrhea

A 4-year old girl of Middle-Eastern Arab origin presented with apparently recessive non-syndromic congenital diarrhea. Exome sequencing of the patient with secondary analysis filtration revealed 590 rare homozygous variants (Additional file 4: Table S3). These were prioritized using VarElect (Fig. 2), showing five gastrointestinally related candidate genes. The strongest phenotype implication for “diarrhea” was for *TTC37*, a known gene for trichohepatoenteric syndrome, harboring a novel homozygous damaging missense mutation in an evolutionary conserved residue, with zero frequency in controls, which withstood validation. Following the detection of this mutation, the patient was confirmed as having atypical syndromic diarrhea. This result emphasize the power of exome sequencing in providing an effective diagnosis for a misdiagnose disease, later found to be an atypical syndrome [30].

### Capillary leak syndrome

Systemic Capillary Leak syndrome (SCLS) is a rare disease characterized by attacks of marked increase in capillary permeability. We identified a first familial SCLS case, an Israeli family with ten affected individuals, showing suspected autosomal dominant inheritance with incomplete penetrance [31]. Exome sequencing was performed for two affected family members (Additional file 5: Table S4). Using VarElect for variant interpretation and submitting 37 post-filtration genes, with “capillary leak” as the phenotype term, no direct hits were obtained (Fig. 3). However, within the 2 top indirect candidates we found *TLN1*, showing a verified severe stop-gain splice mutation, and encoding a cytoskeletal protein that coordinates endothelial dynamics. We hypothesize that the mutated protein could lead, via a dominant-negative model, to aberrant endothelial dysfunction.

### Apnea, Leigh-like

A female patient presented with syndromic apnea and was suspected of having atypical Leigh disease. Other symptoms were brainstem lesion, seizures, mental retardation, nystagmus, bilateral optic atrophy, pulmonary hypertension, and prolonged mechanical ventilation. Following whole exome sequencing of the patient and her two healthy parents (Additional file 6: Table S5), we identified 26 rare homozygous variants in the patient that were heterozygous in both parents and passed other standard filtering criteria. Submitting this gene list to VarElect in relation to the Boolean phenotype expression ‘apnea OR leigh OR brainstem OR seizures OR nystagmus OR “optic atrophy” OR hypertension’, the top gene in direct mode was NDUFAF2, harboring a loss of the start codon [32]. This gene encodes a mitochondrial complex I assembly factor. Mutations in this gene are known to cause Leigh syndrome (MIM 256000), which only in rare cases is associated with infantile apnea [33].

### Arthrogryposis multiplex congenita

An Israeli-Druze family with several members presenting with Arthrogryposis Multiplex Congenita (AMC), characterized by recessive congenital multiple joint contractures, was studied [34]. Following whole-exome sequencing of an affected child, variants remaining post-filtering were subjected to phenotype-driven prioritization (Additional file 7: Table S6). One hundred and fifty six homozygous variants were submitted to VarElect, using “arthrogryposis multiple” as the phenotype. The single direct hit revealed a novel variant in a skeletal muscle protein, with zero frequency in the general population control databases and correctly segregating within the extended family. This is a strong candidate gene for the disease studied.

These four diseases were also subjected to comparative analysis with the four other phenotype interpretation tools, as shown in Table 1.

**Table 1:** Comparative analysis for four diseases with additional phenotype interpretation tools

Target Gene	VarElect		Phenolyzer	Exomiser	Phevor (Omicia)	Ingenuity	
	Direct	Indirect				Direct	Indirect
TTC37	1		3	1	1	77	
MYBPC	1		2	1	1	1	
TLN1		1	0	19	12		58^a^
NDUFAF2	1		1	1	ND	10	

All numbers indicate position in the prioritized list. For Ingenuity, numbers indicate the length of the unprioritized list in which the target gene appears. 0 = not detected; Direct = direct mode for VarElect, 0-hop for Ingenuity; Indirect = indirect mode for VarElect,> 0-hop for Ingenuity; ^a^ = 2-hop; *ND* not done for technical reasons. Diseases associated with the listed gene may be viewed in the “Clinical cases solved by VarElect” section

## Conclusions

NGS currently revolutionizes disease diagnosis and treatment, approaching the point of providing a personal genome sequence for every patient. VarElect, the free web-based Variant Election GeneCards Suite member, addresses a central challenge in this process by performing speedy, comprehensive and highly accurate phenotype prioritization. The integration of VarElect into NGS mapping and filtration pipelines should greatly improve their ability to predict causative mutations. As such, VarElect emerges as an indispensable implement for biomedical interpretation and disease decipherment.

### Consent for publication

Not applicable.

### Ethics approval and consent to participate

All individuals subjected to whole-exome sequencing were recruited either at the pediatric clinic of the Sheba Medical Center in Tel HaShomer, or at the genetic institute at the Rambam health care campus, Haifa, Israel.

The appropriate institutional review boards (regulations for clinical trials in human subjects) approved this research protocol (approval no. 7786-10-SMC). Written informed consent was received from all participants or their guardians.

### Availability of data and material

All appropriate VCF files are included as supplementary data.

## Additional files

Additional file 1: Figure S1. Merging direct and indirect modes. For each tested interlacing factor (x-axis) the red line represents the count of phenotype searches (out of ~300 relevant searches) for which the highest ranking result was direct (left y-axis). The blue line represents the average “interlacing score” (right y-axis) of all relevant phenotype searches. (PDF 52 kb) Additional file 2: Table S1. Gene lists and corresponding phenotypes for Phenolyzer. (XLSX 32.0 kb) Additional file 3: Table S2. Gene lists and corresponding phenotypes for Phenomyzer and Exomiser benchmarking. (XLSX 16.0 kb) Additional file 4: Table S3. Vcf file produced from the exome sequencing of the Congenital Diarrhea clinical case described in the results section. (GZ 14654 kb) Additional file 5: Table S4. Two vcf files produced from the exome sequencing of the Capillary Leak Syndrome clinical case described in the results section, consisting of the inflicted child as well as cousin. (GZ 13018 kb) Additional file 6: Table S5. Three vcf files produced from the exome sequencing of a trio belonging to the Apnea, Leigh-Like clinical case described in the results section. (GZ 13334 kb) Additional file 7: Table S6. Vcf file produced from the exome sequencing of the Arthrogryposis Multiplex Congenita clinical case described in the results section. (GZ 1665 kb)

## Abbreviations

NGS: next generation sequencing
VCF: variant call format
IVA: ingenuity variant analysis
IPA: ingenuity pathway analysis
HPO: human phenotype ontology
MPO: mouse phenotype ontology
DO: disease ontology
GO: gene ontology
BCF: biological context filter
SCLS: systemic capillary leak syndrome
CGA: congenital general anosmia
AMC: arthrogryposis multiplex congenita

## References

[1] Bamshad MJ, Shendure JA, Valle D, Hamosh A, Lupski JR, Gibbs RA, Boerwinkle E, Lifton RP, Gerstein M, Gunel M (2012). The Centers for Mendelian Genomics: A new large-scale initiative to identify the genes underlying rare Mendelian conditions. Am J Med Genet Part A.

[2] Van den Veyver IB, Eng CM. Genome-wide sequencing for prenatal detection of fetal single-gene disorders. Cold Spring Harb Perspect Med 2015, 5(10). doi: 10.1101/cshperspect.a023077 10.1101/cshperspect.a023077 PMC4588135 26253094

[3] Stranneheim H, Wedell A. Exome and genome sequencing: a revolution for the discovery and diagnosis of monogenic disorders. J Intern Med. 2015. 10.1111/joim.12399 26250718

[4] Zheng HF, Forgetta V, Hsu YH, Estrada K, Rosello-Diez A, Leo PJ, Dahia CL, Park-Min KH, Tobias JH, Kooperberg C (2015). Whole-genome sequencing identifies EN1 as a determinant of bone density and fracture. Nature.

[5] Yang Y, Muzny DM, Reid JG, Bainbridge MN, Willis A, Ward PA, Braxton A, Beuten J, Xia F, Niu Z (2013). Clinical Whole-Exome Sequencing for the Diagnosis of Mendelian Disorders. N Engl J Med.

[6] Gilissen C, Hehir-Kwa JY, Thung DT, van de Vorst M, van Bon BWM, Willemsen MH, Kwint M, Janssen IM, Hoischen A, Schenck A (2014). Genome sequencing identifies major causes of severe intellectual disability. Nature.

[7] Smith KR, Bromhead CJ, Hildebrand MS, Shearer AE, Lockhart PJ, Najmabadi H, Leventer RJ, McGillivray G, Amor DJ, Smith RJ (2011). Reducing the exome search space for Mendelian diseases using genetic linkage analysis of exome genotypes. Genome Biol.

[8] Ramos E, Levinson BT, Chasnoff S, Hughes A, Young AL, Thornton K, Li A, Vallania FLM, Province M, Druley TE (2012). Population-based rare variant detection via pooled exome or custom hybridization capture with or without individual indexing. BMC Genomics.

[9] Adzhubei IA, Schmidt S, Peshkin L, Ramensky VE, Gerasimova A, Bork P, Kondrashov AS, Sunyaev SR (2010). A method and server for predicting damaging missense mutations. Nat Meth.

[10] Sim N-L, Kumar P, Hu J, Henikoff S, Schneider G, Ng PC (2012). SIFT web server: predicting effects of amino acid substitutions on proteins. Nucleic Acids Res.

[11] Hecht M, Bromberg Y, Rost B (2015). Better prediction of functional effects for sequence variants. BMC Genomics.

[12] Landrum MJ, Lee JM, Riley GR, Jang W, Rubinstein WS, Church DM, Maglott DR (2014). ClinVar: public archive of relationships among sequence variation and human phenotype. Nucleic Acids Res.

[13] Forbes SA, Beare D, Gunasekaran P, Leung K, Bindal N, Boutselakis H, Ding M, Bamford S, Cole C, Ward S (2015). COSMIC: exploring the world’s knowledge of somatic mutations in human cancer. Nucleic Acids Res.

[14] MacArthur DG, Balasubramanian S, Frankish A, Huang N, Morris J, Walter K, Jostins L, Habegger L, Pickrell JK, Montgomery SB (2012). A systematic survey of loss-of-function variants in human protein-coding genes. Science.

[15] Safran M, Dalah I, Alexander J, Rosen N, Iny Stein T, Shmoish M, Nativ N, Bahir I, Doniger T, Krug H et al. GeneCards Version 3: the human gene integrator. Database (Oxford) 2010, 2010:baq020. doi:10.1093/database/baq020 10.1093/database/baq020 PMC2938269 20689021

[16] Rebhan M, Chalifa-Caspi V, Prilusky J, Lancet D (1997). GeneCards: integrating information about genes, proteins and diseases. Trends Genet.

[17] Rappaport N, Twik M, Nativ N, Stelzer G, Bahir I, Stein TI, Safran M, Lancet D (2014). MalaCards: a comprehensive automatically-mined database of human diseases. Curr Protoc Bioinformatics.

[18] Rappaport N, Nativ N, Stelzer G, Twik M, Guan-Golan Y, Stein TI, Bahir I, Belinky F, Morrey CP, Safran M et al. MalaCards: an integrated compendium for diseases and their annotation. Database (Oxford) 2013, 2013:bat018. doi:10.1093/database/bat018 10.1093/database/bat018 PMC3625956 23584832

[19] Belinky F, Nativ N, Stelzer G, Zimmerman S, Iny Stein T, Safran M, Lancet D: PathCards: multi-source consolidation of human biological pathways. Database (Oxford) 2015, 2015. doi: 10.1093/database/bav006. Print 2015. 10.1093/database/bav006 PMC4343183 25725062

[20] Kononenko O, Baysal O, Holmes R, Godfrey MW (2014). Mining modern repositories with elasticsearch. Proceedings of the 11th working conference on mining software repositories.

[21] Kreuzthaler M, Daumke P, Schulz S (2015). Semantic retrieval and navigation in clinical document collections. Stud Health Technol Inform.

[22] Han JD, Bertin N, Hao T, Goldberg DS, Berriz GF, Zhang LV, Dupuy D, Walhout AJ, Cusick ME, Roth FP (2004). Evidence for dynamically organized modularity in the yeast protein-protein interaction network. Nature.

[23] Yang H, Robinson PN, Wang K (2015). Phenolyzer: phenotype-based prioritization of candidate genes for human diseases. Nat Meth.

[24] Robinson PN, Kohler S, Oellrich A, Sanger Mouse Genetics P, Wang K, Mungall CJ, Lewis SE, Washington N, Bauer S, Seelow D (2014). Improved exome prioritization of disease genes through cross-species phenotype comparison. Genome Res.

[25] Singleton MV, Guthery SL, Voelkerding KV, Chen K, Kennedy B, Margraf RL, Durtschi J, Eilbeck K, Reese MG, Jorde LB (2014). Phevor combines multiple biomedical ontologies for accurate identification of disease-causing alleles in single individuals and small nuclear families. Am J Hum Genet.

[26] Hu H, Huff CD, Moore B, Flygare S, Reese MG, Yandell M (2013). VAAST 2.0: improved variant classification and disease-gene identification using a conservation-controlled amino acid substitution matrix. Genet Epidemiol.

[27] Stelzer G, Dalah I, Stein TI, Satanower Y, Rosen N, Nativ N, Oz-Levi D, Olender T, Belinky F, Bahir I (2011). In-silico human genomics with GeneCards. Hum Genomics.

[28] Edgar R, Mazor Y, Rinon A, Blumenthal J, Golan Y, Buzhor E, Livnat I, Ben-Ari S, Lieder I, Shitrit A (2013). LifeMap Discovery: the embryonic development, stem cells, and regenerative medicine research portal. PLoS One.

[29] Stelzer G, Inger A, Olender T, Iny-Stein T, Dalah I, Harel A, Safran M, Lancet D (2009). GeneDecks: paralog hunting and gene-set distillation with GeneCards annotation. OMICS.

[30] Oz-Levi D, Weiss B, Lahad A, Greenberger S, Pode-Shakked B, Somech R, Olender T, Tatarsky P, Marek-Yagel D, Pras E (2015). Exome sequencing as a differential diagnosis tool: resolving mild trichohepatoenteric syndrome. Clin Genet.

[31] Sion-Sarid R, Lerman-Sagie T, Blumkin L, Ben-Ami D, Cohen I, Houri S (2010). Neurologic involvement in a child with systemic capillary leak syndrome. Pediatrics.

[32] Zhu X, Petrovski S, Xie P, Ruzzo EK, Lu YF, McSweeney KM, Ben-Zeev B, Nissenkorn A, Anikster Y, Oz-Levi D (2015). Whole-exome sequencing in undiagnosed genetic diseases: interpreting 119 trios. Genet Med.

[33] Shuk-kuen Chau C, Kwok KL, Ng DK, Lam CW, Tong SF, Chan YW, Siu WK, Yuen YP (2010). Maternally inherited Leigh syndrome: an unusual cause of infantile apnea. Sleep Breath.

[34] Ekhilevitch N, Kurolap A, Oz-Levi D, Mory A, Hershkovitz T, Ast G, Mandel H, Baris HN. Expanding the MYBPC1 phenotypic spectrum: a novel homozygous mutation causes arthrogryposis multiplex congenita. Clin Genet. 2015. doi: 10.1111/cge.12707. 10.1111/cge.12707 26661508

