# Supplementary figures and images for: VarElect: the phenotype-based variation prioritizer of the GeneCards Suite
Source: BMC Genomics. 2016 Jun 23;17(Suppl 2):444. doi: 10.1186/s12864-016-2722-2 (PMC4928145; doi:10.1186/s12864-016-2722-2)

Figure S1

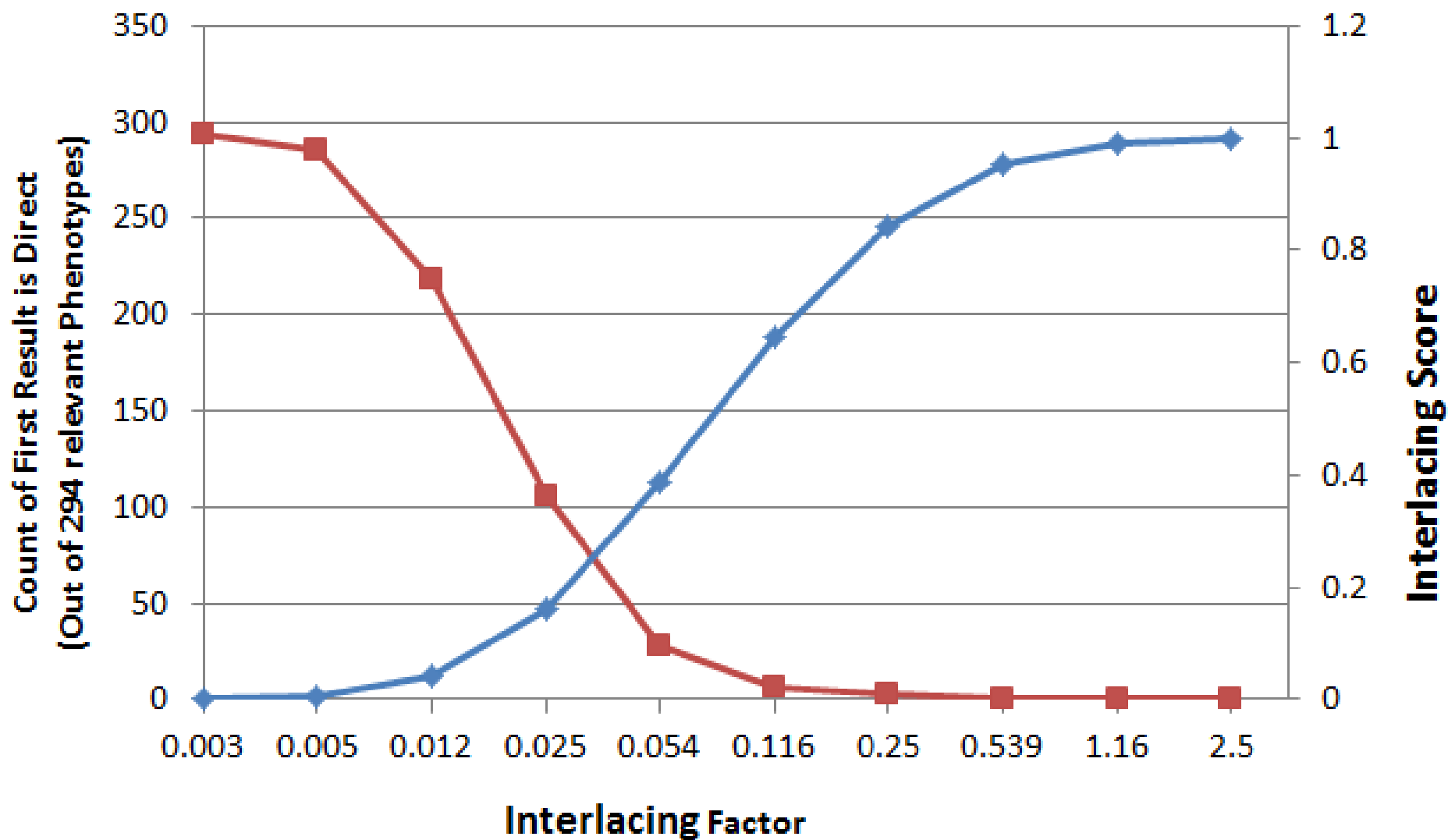

Supplement: Additional file 1: Figure S1. — Merging direct and indirect modes. For each tested interlacing factor (x-axis) the red line represents the count of phenotype searches (out of ~300 relevant searches) for which the highest ranking result was direct (left y-axis). The blue line represents the average “interlacing score” (right y-axis) of all relevant phenotype searches. (PDF 52 kb) [file 12864_2016_2722_MOESM1_ESM.pdf]
